# High Accuracy Human Activity Recognition Based on Sparse Locality Preserving Projections

**DOI:** 10.1371/journal.pone.0166567

**Published:** 2016-11-28

**Authors:** Xiangbin Zhu, Huiling Qiu

**Affiliations:** College of Mathematics, Physics and Information Engineering, Zhejiang Normal University, Zhejiang, China; West Virginia University, UNITED STATES

## Abstract

Human activity recognition(HAR) from the temporal streams of sensory data has been applied to many fields, such as healthcare services, intelligent environments and cyber security. However, the classification accuracy of most existed methods is not enough in some applications, especially for healthcare services. In order to improving accuracy, it is necessary to develop a novel method which will take full account of the intrinsic sequential characteristics for time-series sensory data. Moreover, each human activity may has correlated feature relationship at different levels. Therefore, in this paper, we propose a three-stage continuous hidden Markov model (TSCHMM) approach to recognize human activities. The proposed method contains coarse, fine and accurate classification. The feature reduction is an important step in classification processing. In this paper, sparse locality preserving projections (SpLPP) is exploited to determine the optimal feature subsets for accurate classification of the stationary-activity data. It can extract more discriminative activities features from the sensor data compared with locality preserving projections. Furthermore, all of the gyro-based features are used for accurate classification of the moving data. Compared with other methods, our method uses significantly less number of features, and the over-all accuracy has been obviously improved.

## Introduction

With the rapid development of information technology, it has been observed there is an accelerated growth of smartphones, which incorporate a variety of sensors, such as high-resolution cameras, light sensors, gyroscopes, accelerometers, GPS, temperature sensors and so on [1]. In recent years, the people with smartphones have reached near 80% of the world adult population [2], and by 2020, it will reach 80% [3]. It can be envisioned that such powerful devices can provide a tool to automatically monitor activities of daily living (ADL) and enhance us the ability of making better decision regarding our future actions [4]. This is not only for its flexibleness, convenience and availableness but also for its easiness to use [5]. For these reasons, human activity recognition with smartphone sensor data has been a hot research topic.

There have been a lot of researches to identify human activity based on video data. Many of these results can be applied to human activity recognition of sensor data. But we need to take full account of the features of smartphone sensor data. Firstly, smartphone sensor data is multi-sensor data streams. The features of smartphone sensor data usually have more dimensionality than that of video data in human activity recognition. Secondly, not like HAR of video data, HAR of smartphone sensor data cannot directly recognize a human activity from shape features, which can be extracted from video data. HAR of smartphone sensor data need to indirectly infer the human activity from sensor data streams, such as accelerometers data, gyroscopes data and localization sensor data. Therefore, we should take advantage of the characteristics of human activity. For example, we can classify human activity based on the characteristics of human activity so that the common attribute of one category of human activity can be employed by HAR. Moreover, human activities can be hierarchically classified because human activities can be grouped into subsets as cluster analysis does. Thus, we can hierarchically recognize human activity with a multi-stage classifier.

Nowadays, the recognition of human activity using smartphones sensor has an upsurge of interest for many researchers and gained preliminary achievements. For example, there have been proposed some outstanding AI methods on human recognition. Wei *et al*. [6] used a hidden Markov model(HMM) to build the cloud resource allocation model, which is based on an imperfect information Stackelberg game. The HMM method was employed to predict the service provider’s current bid. Li *et al*. [7] proposed a conditional random field based event detection method to analyze the human group behavior, and the human groups were identified by a temporal-spatial clustering approach. Lee and Cho proposed hierarchical hidden Markov models(HHMMs) [8], Sharma *et al*. used neural network [9], Anguita *et al*. used Multiclass Hardware-Friendly Support Vector Machine [10] and Ronao *et al*. introduced the technology of two-stage CHMMs [11]. However, these research results are not effective enough for some applications. So, we need a creative method to improve performance in several aspects including accuracy and speed. In dataset processing, for instance, dimensionality reduction, which aims to reduce the number of features, has still room for improvement. For example, Anguita *et al*. used 561 variables and Charissa Ann Ronao *et al*. selected 119 variables. Moreover, although many different classification methods have been used, the final recognition rate is still not accurate enough, especially for the easily confused activities. One reason is the different physical location of sensor which volunteer worn to perform the activities might cause different data collected [5], and the data may be similar with features so that models are difficult to distinguish them.

Our approach is combined with dimensionality reduction and hierarchical classifier. Dimensionality reduction includes feature selection and feature reduction. Since the time series data we used in the experiment is continuous and stanardized, each data point is very closely related with adjacent data points. Random forests variable importance measures perform well with these data [12]. The obtained database is real data sets and randomly partitioned into two sets: testing set and training set. Moreover, these data are high correlation coefficient of local, particularly in the stage of feature reduction in which the data local correlation coefficient is higher than the data in feature selection stage. SpLPP(Sparse Local Preserving Projections) optimally preserves the neighborhood structure the data set. It can choose the number of neighborhood and calculates the weight value adaptively. Moreover, SpLPP can extract more discriminative activities features from the sensor data compared with locality preserving projections. The variables of gyroscope play a leading role for stationary activities and the acceleration variables play a minor role [11]. Thus, we choose random forests variable importance measures as the method of feature selection and sparse local preserving projection as the way of feature reduction.

The accelerometer and gyroscope sensory data are sequential nature and multivariate. This is advantageous to use the CHMM to classify because those characteristic are properly consistent with the nature of this method [13]. These activities are hierarchical, which means we can multiple utilize the method of “one divides into two” to recognition activities. In this paper, we use a three-stage CHMMs approach.

## Materials

In this section, we present a three-stage continuous hidden Markov model (TSCHMM) approach, which use random forests variable importance measures for feature selection and sparse local preserving projection for feature reduction. Meantime the three-stage CHMM is advantageous to the sensory data which has typical temporal characteristics and the inherent hierarchical frame of activities.

**Fig 1:**
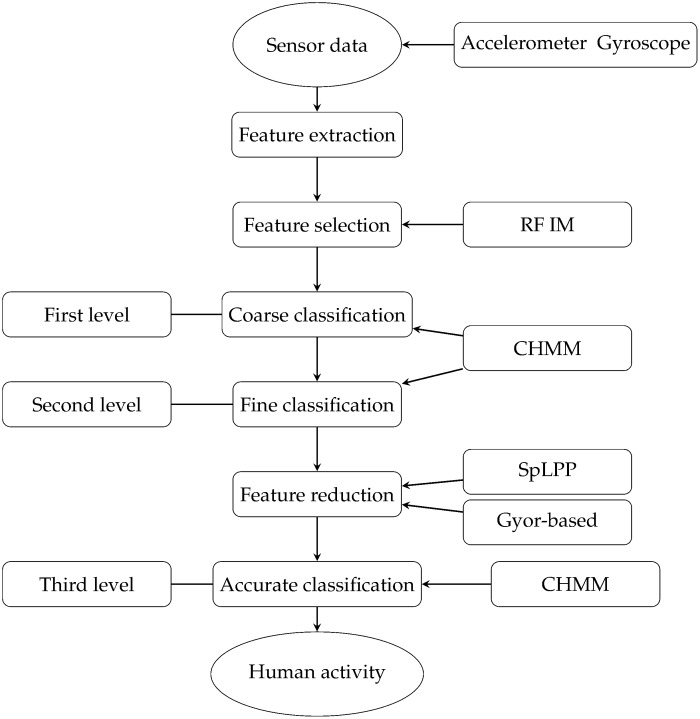
Human Activity Recognition Process Hierarchy.

Our method is shown in Fig 1. The signal data is collected by the acceleration and gyroscopic sensor in a smartphone. In this paper, for introducing our work, we focus on six basic human daily activities, including Walking, Upstairs, Downstairs, Sitting, Standing and Laying. The first step is feature extraction. We totally extracted 561 features to describe each activity window, including the standard measures: means, correlation, signal magnitude area (SMA), autoregression coefficients and the new features: energy of different frequency bands, frequency skewness, angle between vectors. The next is feature selection, using the RF to compute their importance scores. At coarse classification stage, TSCHMM divides the feature subset into moving subclass and stationary subclass. Moving subclass includes Walking, Upstairs and Downstairs. Stationary subclass includes Sitting, Standing and Laying. Different subclasses correspond to their respective feature subsets. At the second classification stage, the fine classifier classifies the moving subset into Walking subclass and Up-Downstairs subclass, the stationary activity is divided into Laying sub-activity and Sitting-Standing sub-activity. Then, the next step is feature reduction, using the SpLPP and Gyro-based to reduce the number of features. TSCHMM for accurate classification divides the final feature subsets into subclasses of Sitting, Standing, Upstairs and Downstairs.

### Random Forest Importance Measures(RFIM)

Dimension reduction need not only to reduce the amount of data but also to find out which features are more important for each activity. The key technology is to quantitatively evalute the importance of each feature. This paper employs the random forest importance measures method, which can effectively estimate each varialbe importance.

Random forests is a powerful ensemble method for classification, introduced by L. Breiman(2001). It builds a set of de-correlated trees [14]. RF(random forests) is a classifier composing of simple decision trees:

$$\begin{array}{c} k(x,\Theta_{h}),h=1,...n; \end{array}$$ (1)

where {Θ_*h*_} are i.i.d random vectors and each tree casts a unit vote for the most popular class at input *x*.

This algorithm combines the idea of Boostrap aggregation and bagging technology, and it has been proved, when dealing with a lot of redundant features problems, that bagging is worse than random subspace selection [15]. This method also has other important features such as out-of bag (OOB) error, variable importance and correlation.

In this paper, we emphasize two different objectives about variable selection: (1) to find out important variables which highly related to the response variable for explanation purpose; (2) to find out a small number of data volume for a good prediction of the response variable [16]. Let us briefly show the experiment framework:

1. Get the RF variable importance scores *a*_*i*_, *i* = 1, …, *n*;
2. Order the *n* variables in decreasing;
3. Compute the averages scores *b* of RF variable importance scores;
4. Remain the variables *a*_*i*_, *a*_*i*_ ≥ *b*, *i* = 1, …*n*, which scores are above the average;

### Sparse Locality Preserving Projections(SpLPP)

According to the data type, the data dimension reduction methods can be divided into linear and nonlinear dimensionality reduction [17]. Typical linear dimension reduction algorithms include Principal Component Analysis (PCA), Multidimensional Scaling (MDS) and Linear Discriminant Analysis (LDA). Similarly, there are several nonlinear dimensionality reduction(Manifold Learning) methods, such as Isometric map (Isomap), Locally Linear Embedding (LLE), Laplacian Eigenmaps (LE), Local Tangent Space Alignment (LTSA) and Locality Preserving Projections (LPP). Some algorithms such as PCA and LDA have solid theoretical foundations and they are easy to analyze. However, these methods also ignore the intrinsic geometry structural features of high dimensional space and finally lead to the low recognition rate. The manifold learning methods such as LLE, Isomap and LE exhibit good performance on some artificial data set, but on the real data set, they cannot get better results and often lead to “out-of-sample” problem.

LPP is a popular and efficient algorithm for linear manifold learning. It preserves the advantages of the nonlinear manifold learning and linear dimensionality reduction method [18]. Moreover, LPP has overcome the “out-of-sample” problem and develops the performance of manifold learning on real data. LPP establishs the data mapping base on neighborhood graph and keeps the local structure of data, then makes the high dimensional data X mapped to a low dimensional data Y, such that the points which are close in the higher dimensional space will be still mapped as close in lower dimensional space [19]. LPP aims to seek a transformation matrix *A* to implement the dimensionality reduction of data; that is *y*_*i*_ = *A*^*T*^ *x*_*i*_. The objective function:

$$\begin{array}{c} J=\sum_{i=1}^{n}\sum_{j=1}^{n}\|y_{i}-y_{j}{\|}^{2}W_{ij}; \end{array}$$ (2)

which *W*_*ij*_ are the projection weights between samples *x*_*i*_ and samples *x*_*j*_.

We can get the transformation matrix *A* by minimizing the function as following algebraic steps:

$$\begin{array}{c} A_{opt}=argmin\left(\sum_{i=1}^{n}\sum_{j=1}^{n}\|y_{i}-y_{j}{\|}^{2}W_{ij}\right)=\\ argmin(\sum_{i=1}^{n}\sum_{j=1}^{n}\|A^{T}x_{i}-A^{T}x_{j}{\|}^{2}W_{ij})=\\ argmintr(A^{T}XDX^{T}A-A^{T}XWX^{T}A)=\\ argmintr(A^{T}XLX^{T}A) \end{array}$$ (3)

where D is the diagonal matrix, $D_{ii}=\sum_{j=1}^{n}W_{ij}$;*L* = *D* − *W* is the Laplacian matrix, *W* is the projection weight matrix comes from *W*_*ij*_.

In order to remove the arbitrary scaling factor, we impose a constraint condition *YDY*^*T*^ = 1. The transformation matrix can be obtained by solving the generalized eigenvalue problems:

$$\begin{array}{c} XLX^{T}A=\lambda XDX^{T}A \end{array}$$ (4)

Let the solutions of the Eq 2 be the column vector *a*_1_, *a*_2_, …, *a*_*d*_, sorted according to their eigenvalue *λ*_1_, *λ*_2_, …, *λ*_*d*_. Then we can get *A* = (*a*_1_, *a*_2_, …, *a*_*d*_). Thus, the embedding is as follows:

$$\begin{array}{c} x_{i}\to y_{i}=A^{T}x_{i},A=\left(a_{1},a_{2},...,a_{d}\right); \end{array}$$ (5)

where *y*_*i*_ is the d-dimensional vector and *A* is a matrix of n*d.

The drawback of local preserving projection algorithm is the number of samples which are contained in the local neighborhood cannot be adaptively selected. Moreover the neighborhood graph is very sensitive to data noise. However, SpLPP can effectively combine the LPP with the Sparsity representation theory. It gets the optimal solution of sparse representation by using *l*_1_ norm method in the LPP algorithm [20] and solves the parameters selection. More importantly, it achieves the purpose of keeping the reconstruction relationship of input data.

SpLPP algorithm aims to use the sparsity representation theory to construct the projection weight *W*_*ij*_. For each sample *x*_*i*_, we compute the *l*_1_ norm problem to find out its sparsity reconstruction weight coefficient *W*_*i*_.

$$\begin{array}{c} min_{w_{i}}\|x_{i}-Xw_{i}{\|}_{2}+\lambda {\left\|w_{i}\right\|}_{1};w_{i,j}\geq 0,j=1,2,...,m; \end{array}$$ (6)

where [*w*_*i*,1_, …, *w*_*i*, *i* − 1_, 0, *w*_*i*,*i*+1_, …, *w*_*i*, *m*_]^*T*^ is a m-dimensional column vector and the i-th is 0, the other elements *w*_*i*,*j*_, *j* ≠ *i* denote the contribution of each sample *x*_*j*_ to reconstruction the sample *x*_*i*_.

By calculating the weight vector of each sample, we finally get the sparse reconstruction weight matrix *W* = (*w*_*i*,*j*_)_*m* × *m*_, which *W*_*i*_ is the optimal solution of sparse representation.

With the reduced dimension and local variance information preservation, the extracted features y will be used as the new input features for accurate recognition [21].

### Continuous Hidden Markov Models(CHMM)

Hidden Markov models(HMM) arises out by a simple Markov chain. It is a statistical model for sequence of data items named observation vectors and system states cannot be observed but obtained through observation vector. It is an important method for both automatic speech recognition (ASR) and signal processing [22]. HMMs are based on a hidden Markov chain {*S*_*n*_}:the state transition of the system. A sequence of hidden state {*S*_*n*_} and the observed variables {*O*_*n*_} are conditionally independent. Each of the distribution *O*_*n*_ only relies on the corresponding state *S*_*n*_. Besides, HMM is a double stochastic procedure: the transitions between states; the statistical relationship between state and observation symbol. Since state-conditioned observation distributions of sensory data is continuous, we use the continuous HMMs in this paper. CHMMs are composed of two state sets and three probability matrices, the model as following equation:

$$\begin{array}{c} \lambda =(A,B,\pi ); \end{array}$$ (7)

1. Hidden state *S*: the state is satisfied with Markov property and it is the actual state of the hidden Markov model;
2. Observation state *O*: the state is associated with the hidden state and it is observable;
3. Initial state distribution *π*: the distribution denote the hidden state probability matrix when *t* = 1,
  $$\begin{array}{c} \pi_{i}=P\left(S_{0}=i\right),1\leq i\leq N; \end{array}$$ (8)
  where *N* is the probability distribution number;
4. Transition probability matrix *A*: the matrix describe the transition probability between hidden states,
  $$\begin{array}{c} a_{ij}=P\left[S_{t+1}=j|S_{t}=i\right],1\leq i,j\leq N; \end{array}$$ (9)
5. Observation probability matrix *B*: the matrix is represent the transition probability between hidden state and observation state,
  $$\begin{array}{c} b_{i}\left(o\right)=p_{o}\left(o;\theta_{i}\right)=f\left(o;\theta_{i}\right); \end{array}$$ (10)
  where *p*_*o*_(*o*; *θ*_*i*_) indicate the emission density of state *i*,
  $$\begin{array}{c} p_{o}\left(o;\theta_{i}\right)=\frac{1}{\sqrt{2\pi \sigma_{i}}}exp-\frac{{\left(o-\mu_{i}\right)}^{2}}{2\sigma_{i}^{2}}; \end{array}$$ (11)
  Eq 11 is a Gaussian distribution, the mean is *μ* and variance is *σ*^2^.

### Three-Stage Continuous Hidden Markov Models(TSCHMMs)

Three-Stage Continuous Hidden Markov Models (TSCHMMs) exploit the hierarchical nature of activities and the arithmetical of CHMM. The recognition process starts with the acquisition of the sensor data. It contains coarse, fine and accurate stage classification which are described in the following section.

#### The First-Stage CHMMs for Coarse Classification

The sensor data, including six daily living activities, was gathered from the sensors built in a smartphone. In the first stage, we classify six activities into two classes (moving and stationary). As Alg. 1 shows, the sensor data needs to be preprocessed, be processed for features extraction by RFIM and then be supplied to CHMMs for first-level training and testing. Moving train data is used to train moving CHMM and stationary train data is fed to train stationary CHMM. The feature subsets are preselected and used in each step.

**Algorithm 1 First-stage CHMMs for Classification**.

**Input:** *X* train: train data; *y* train: train label; *X* test: test data; *y* test: test label; *O*: Number of coefficients in a vector; *Q*: Number of states; *M*: Number of mixtures;

**Output:** Stationary, Moving

1:  Compute the RFVI scores *a*_*i*_ and the averages scores *b*

2:  **while** *i*← 1 *to* t **do**

3:  **if** *a*_*i*_ ≥ *b* **then**

4:   *i*1 ← *i*1 + 1

5:   *data*(:,i1) ← *Xtrain*(:,i)

6:  **else**

7:   delete *Xtrain*(:,i)

8:  **end if**

9:  **end while**

10: **while** *j*← 1 *to* n **do**

11:  **if** *y* train (*j*) in {1 6} **then**

12:   *Xtrain*1(j,:) ← *data*(1, *j*1,:)

13:  **else**

14:   *Xtrain*2(j,:) ← *data*(1, *j*2,:)

15:  **end if**

16: **end while**

17: Training Laying CHMM or Training Walking CHMM

18: **for** *k*← 1 *to* m **do**

19:  *Xtest*(k,:) ← *data*(1, 1,:)

20:  compute the model log-likehood

21: **end for**

22: Selection through max probability

When train data has been trained moving CHMM and stationary CHMM, test data is supplied to the two CHMMs. The result can get two probability values to assess the classes. Thus if moving CHMM probability value is higher than stationary CHMM probability value, we can classify that it is the class of moving, vice versa. Hence, setting a subclass *r* ∈ *R*, we can build a CHMM *λ*^*r*^, then evaluate the initial state distribution *π*, transition probability matrix *A* and observation probability matrix *B*, finally optimize the corresponding training state likelihood.

Based on the three basic problems and the corresponding algorithms of HMM [22] [23], in the first-stage testing, we use the feature subset *O* to estimate the observation likelihood of the subclass *r* ∈ *R*. We use the forward-backward algorithm to compute all of CHMMs for each subclass by *P*(*O*∣*λ*^*r*^) and then get the CHMM of each subclass. This evidently requires a method to allow comparability between each CHMM. For this reason, we have opted to compute log-likelihood using a (mixture of) Gaussians HMM for each subclass and to choose the activity which corresponding to the highest probability, the relationship as:

$$\begin{array}{c} r^{{}^{\prime }}=argmax_{r\in R}P\left(O|\lambda^{r}\right); \end{array}$$ (12)

#### The Second-Stage CHMMs for Fine Classification

Each activity has its unique property. The Laying activity can be very efficiently classified from stationary class [24]. But for the subclass of moving, the state of Walking can be recognized as upstairs or downstairs with high probability. As Alg. 2 shows, the purpose of the second-stage CHMMs, combined with the activity hierarchical nature, is to recognize the Walking and Laying. In order to achieve this, based on the first-stage data, the process goes on running either towards the Laying subclass or the Sitting-Standing subclass. That means Laying train data is fed to the Laying HMM, Sitting-Standing train data is fed to Sitting-Standing HMM. As the same way, data of Walking is used to be trained Walking HMM, the same as data of Up-Downstairs. Finally, stationary and moving subclass will be classified into 2 activities.

**Algorithm 2 Second-stage CHMMs for Classification**.

**Input:** *X* train: train data; *y* train: train label; *X* test: test data; *y* test: test label; *O*: Number of coefficients in a vector; *Q*: Number of states; *M*: Number of mixtures;

**Output:** Laying, Sitting-Standing, Walking, Up-Downstairs

1:  **while** *j*← 1 *to* n **do**

2:  **if** *y* train (*j*) in {1 6} **then**

3:   *Xtrain*1(j,:) ← *data*(1, *j*1,:)

4:  **else**

5:   *Xtrain*2(j,:) ← *data*(1, *j*2,:)

6:  **end if**

7:  **end while**

8:  Training Laying CHMM or Training Walking CHMM

9:  **for** *k*← 1 *to* m **do**

10:  *Xtest*(k,:) ← *data*(1, 1,:)

11:  compute the model log-likehood

12: **end for**

13: Selection through max probability

At this stage, we can build CHMM *λ*^*rs*^ for each stationary activity. Same as the first level, we get the highest probability $r_{s}^{{}^{\prime }}$ for stationary subclasses, individually. We also build CHMM *λ*^*rm*^ for each moving activity and get the highest probability $r_{m}^{{}^{\prime }}$ for moving subclasses. Noteworthy, the number of mixtures *M* is changed.

#### The Third-Stage CHMMs for Accurate Recognition

The last stage is, as shown in Alg. 3 and Alg. 4, aims to recognize Sitting and Standing from the subclass of Sitting-Standing and to classify Upstairs and Downstairs from Up-Downstairs data. The training and testing process will continue to run as the first and second level. We use two states for CHMMs and each of the subclass will be classified into two activities.

**Algorithm 3 Third-stage CHMMs for Sitting-Standing classification**.

**Input:** *X* train: train data; *y* train: train label; *X* test: test data; *y* test: test label; *O*: Number of coefficients in a vector; *Q*: Number of states; *M*: Number of mixtures;

**Output:** Standing, Sitting

1:  *y*_*i*_ = *A*^*T*^ *x*_*i*_

2:  **while** *j* ← 1 *to n* **do**

3:  **if** *y* train (*j*) in {4 5} **then**

4:   *Xtrain*1(j,:) ← *data*(1, *j*1,:)

5:  **else**

6:   *Xtrain*2(j,:) ← *data*(1, *j*2,:)

7:  **end if**

8:  **end while**

9:  Training Standing CHMM or training Sitting CHMM

10: **for** *k*← 1 *to* m **do**

11:  *Xtest*(k,:) ← *data*(1, 1,:)

12:  compute the model log-likehood

13: **end for**

14: Selection through max probability

**Algorithm 4 Third-stage CHMMs for Up-Downstairs classification**.

**Input:** *X* train: train data; *y* train: train label; *X* test: test data; *y* test: test label; *O*: Number of coefficients in a vector; *Q*: Number of states; *M*: Number of mixtures;

**Output:** Upstairs, Downstairs

1:  **while** *i*← 1 *to* t **do**

2:  **if** feature is Gyro-based data **then**

3:   *i*1 ← *i*1 + 1

4:   *data*(:,i1) ← *Xtrain*(:, i)

5:  **else**

6:   delete *Xtrain*(:,i)

7:  **end if**

8:  **end while**

9:  **while** *j*← 1 *to* n **do**

10:  **if** *y* train (*j*) in {2 3} **then**

11:   *Xtrain*1(j,:) ← *data*(1, *j*1,:)

12:  **else**

13:   *Xtrain*2(j,:) ← *data*(1, *j*2,:)

14:  **end if**

15: **end while**

16: Training Downstairs CHMM or training Upstairs CHMM

17: **for** *k*← 1 *to* m **do**

18:  *Xtest*(k,:) ← *data*(1, 1,:)

19:  compute the model log-likehood

20: **end for**

21: Selection through max probability

As previously mentioned, we build CHMM *λ*^*rss*^ for each Sitting-Standing activity and *λ*^*rmm*^ for each Up-Downstairs activity, then estimate the model parameter (*A*, *B*, *π*)_*ss*_ and (*A*, *B*, *π*)_*mm*_, finally optimize the corresponding training states likelihood. Just as the previous levels, we get the highest probability $r_{ss}^{{}^{\prime }}$ and $r_{mm}^{{}^{\prime }}$ for each subclass, individually.

It is worth noting that the feature of subclass in this level will be processed. We use SpLPP for feature reduction to get the new feature subsets which are fed to both Sitting and Standing CHMMs. For another group, basing on the original sensor data, we remain the gyroscope variables and remove the acceleration variables to obtain new subset which are used to train Upstairs CHMM and Downstairs CHMM.

## Results

In this paper, the experiment data is from the public domain UCI HAR data set, including accelerometer and gyroscope XYZ data [25]. The related sensors incorporate in a Samsung Galaxy SII smartphone, which was worn by each volunteer. Each person performed the six activities (WALKING, UPSTAIRS, DOWNSTAIRS, SITTING, STANDING, LAYING) twice. Every time the phones were worn on different location. One was on the left-side of the belt and the other was free setting by users. The sensor signals (accelerometer and gyroscope) were pre-processed by applying noise filters and then sampled in fixed-width sliding windows of 2.56 sec and 50% overlap(128 readings/ window). Thus, 17 signals were totally obtained by calculating variables in the time and frequency domain (e.g. mean, standard deviation, signal magnitude area, entropy, signal-pair correlation, ect.). Each activity window was described by 561 features and normalized between -1 and 1. Through the randomly partition, 70% of the volunteer dataset was selected as training data and 30% of the dataset was selected as testing data [25].

The idea of using RFIM feature selection proposed by the Genuer, et. al [16]. We ran repeat RF tests (*ntree* = 500;*mtry* = *sqrt*(*size*(*X*, 2))) and got all of the variable importance scores. Then we ranked the scores in descending order and computed the average of all the variable importance scores, finally remained the variables which scores are above the average. After these processes, we kept 132 variables as the first stage feature subset. Moving and stationary feature subsets in the second stage are from the first stage feature subset.

We adopt the method of SpLPP feature reduction similar to the paper of Zheng Z, et. al. [20]. For subclass of Up-Downstairs on the second level feature subset, the number of the related features could be reduced from 132 to 13 by SpLPP technology. The 13 features would be used in the third stage. Based on original data, we selected the gyroscope variables and gave up the acc-based variables. Thus, the number of the related features was reduced from 561 to 218 and the 218 featuers would be used as third level subset.

**Fig 2:**
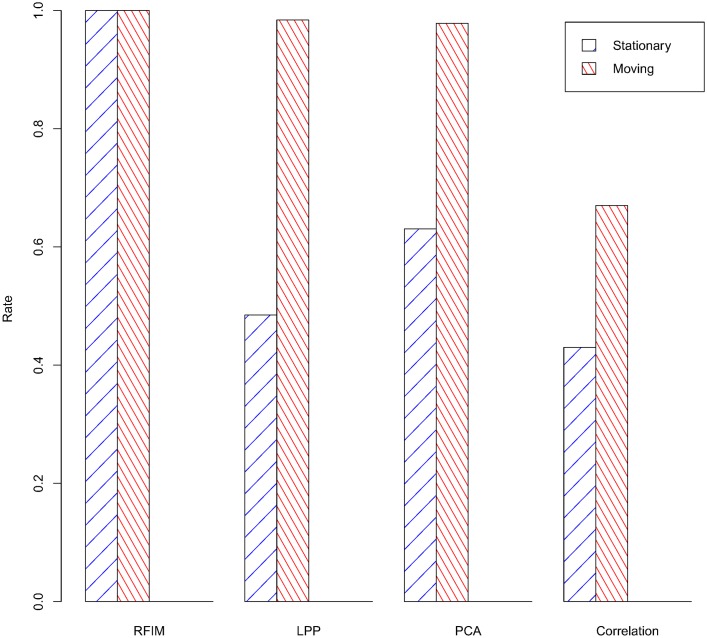
Coarse Classification Rate of Different Feature Selection Techniques.

**Fig 3:**
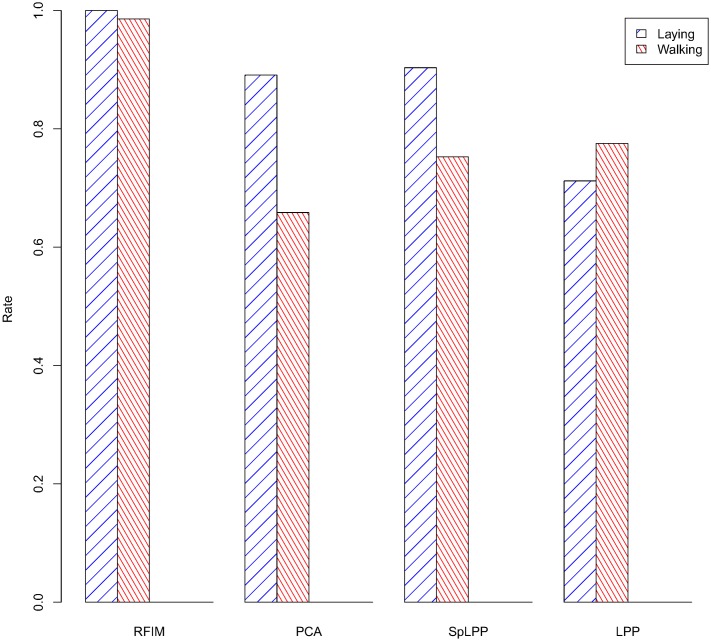
Fine Classification Rate of Different Feature Selection Techniques.

**Fig 4:**
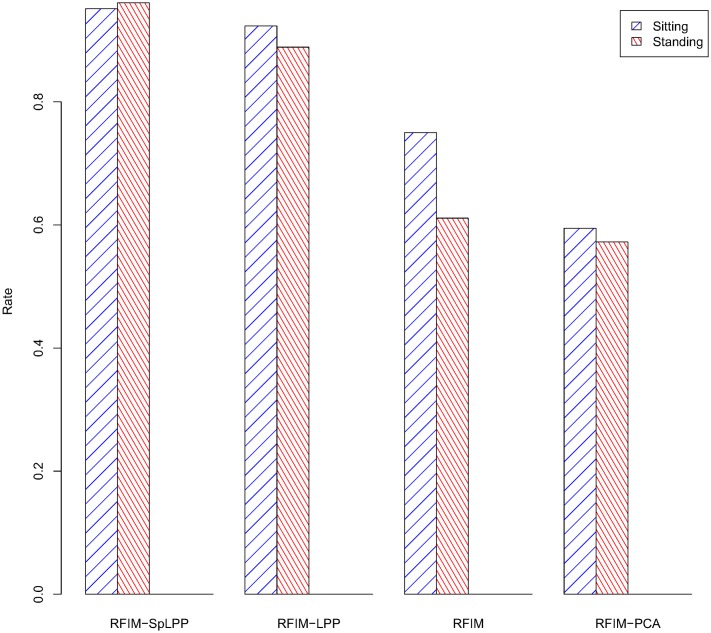
Accurate Classification Rate of sitting and standing activities.

**Fig 5:**
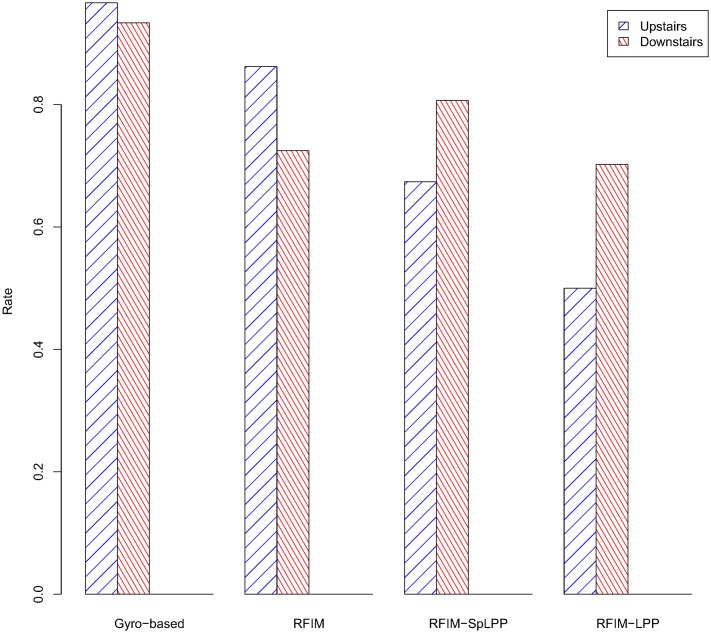
Accurate Classification Rate of upstairs and downstairs activities.

For evaluating the performance of the RFIM-SpLPP, a set of experiments were carried out using the HAR dataset as mentioned before. They consisted of learning CHMMs with different feature selection techniques and then comparing their performance in terms of test data error. In order to test the performance of our method, in the experiments, we used many dimension reduction algorithms, such as RFIM, PCA, LPP, Correlation and SpLPP. As we can see from Fig 2, in the first stage, we used RFIM (132 features), LPP (118 features), PCA (118 features) and Correlation (232 features) to reduce the dimension of sensor data. The reult is that the RFIM performed well than others. At the second stage, we compared the methods of RFIM (132 features), PCA (118 features), SpLPP (97 features) and LPP (118 features). From the Fig 3 and we can be easy to see that RFIM (132 features) lead to the better performance. At the third stage, for the different activities, we selected the different dimension reduction methods (Gyro-baesd(218 features), RFIM-SpLPP(13 features), RFIM-LPP(25 features), RFIM-PCA(25 features)) and we can see that the accuracy rate of the Gyro-based and RFIM-SpLPP are significantly higher than that of other methods. The detailed result of these comparison are depicted in Figs 4 and 5.

We show the confusion matrix of the classification results on test data using TSCHMMs in Table 1. The table evidently reveals that for this classifier of TSCHMMs is sufficient for achieving optimum performance. The test error remains stable and our method for the Walking activity and the Laying activity has high-performing, especially for Laying activity for which the performance achieved complete proper recognition. Moreover, it is also seen from the table that Sitting was the most difficult activity for classification because the physical location of Sitting is between the position of Laying and Standing, thus it is easy to be recognized as other two activities. It also shows that latter activities are more easily confused and the main cause might be the physical location of sensor where volunteer worn to perform the activities.

**Table 1:** Confusion Matrix of Classification Results on Test Data Using TSCHMMs.

	walking	upstairs	downstairs	sitting	standing	laying	precision
walking	489	7		0	0	0	98.59%
upstairs	0	455	16	0	0	0	96.60%
downstairs	0	28	392	0	0	0	93.33%
sitting	0	0	0	467	24	0	95.11%
standing	0	0	0	21	511	0	96.07%
laying	0	0	0	0		537	100%

**Fig 6:**
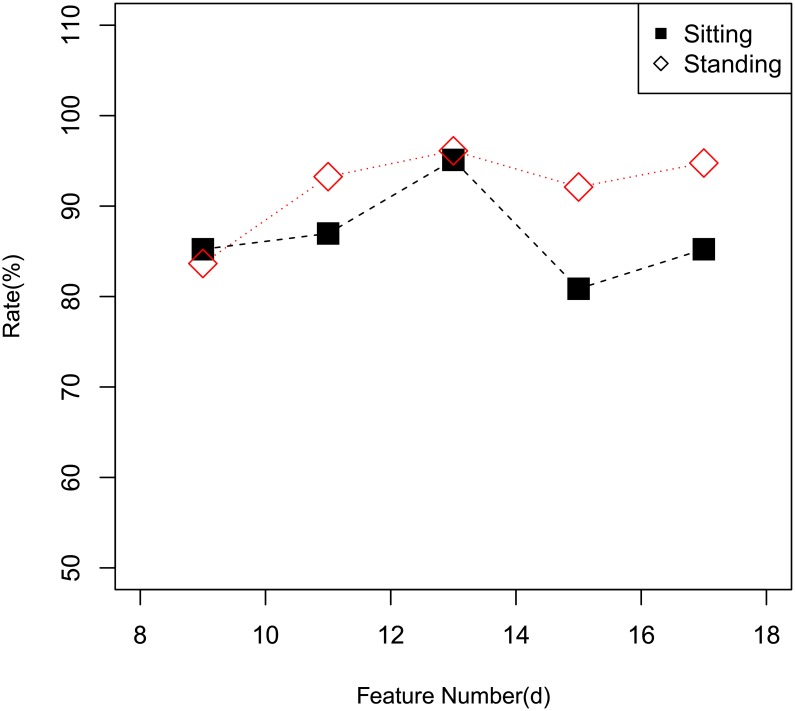
Accuracy Rate of SpLPP with Different Feature Number.

The Fig 6 shows the recognition results with different number of features obtained from the SpLPP. We can see that there is a large difference in the accuracy rate of different features number, and the reason is: SpLPP optimally preserves the geometric features of the original data, if *d* is large, the mapping result will contain too much noise, similarly, if *d* is small, the different points may overlap in the low dimensional space. So in our simulation experiment, 13 is the optimal feature number.

We have compared the proposed method with many other statistical techniques such as RF, conventional HMM and Two-level HMM. The classification result of those approaches is depicted in Table 2, where estimates of the precision is given. 132 features were used to evaluate the performance of those methods. The figure illustrates that the accuracy of third-stage CHMMs and two-stage CHMMs vary slightly for Laying activity, Standing activity and Walking activity. But for the Downstairs activity, the Upstairs activity and Sitting activity, the third-stage CHMMs perform better. Therefore, our proposed method of TSCHMM is more competitive with the other algrothims.

**Table 2:** Resulting Recognition Rates of Different Classifiers.

	Third-stage CHMM	Two-stage CHMM	HMM	RF
Laying	100%	99.33%	94.79%	100%
Standing	96.07%	96.43%	77.07%	90.41%
Sitting	95.11%	90%	44%	85.34%
Down-stairs	93.33%	86.24%	81.43%	84.52%
Up-stairs	96.6%	92.36%	77.71%	89.81%
Walking	98.59%	96.13%	87.1%	97.18%

## Discussion

It is novel and effective for the three-stage continuous hidden Markov model method to recognize the human daily activities based on smartphone sensor data. We have taken into account of the human activity’s characteristics and propose the innovative combination method for human activity recognition. The TSCHMMs use less number of features and obtain evident high accuracy. From our simulation study, it is observed that as the strength of the relationship between the dimensionality reduction and hierarchical classifier, the performance of our method combined with dimensionality reduction and hierarchical classifier is considerably and consistently better than the ones only using classifier. In our method, we have chosen the RFIM-SpLPP technology. In our expriments, RFIM-SpLPP was compared with the method only using RFIM or SpLPP. The result is that RFIM-SpLPP is indeed the better. Hence, with appropriate reduced feature set, the TSCHMMs can obtain the equivalent classifier performance and outstanding recognition rate.

We have examined the result of the classification performance for each class. This is one key guarantee that the probability of moving activity appearing in the stationary subset or stationary activity existing in the moving subset is 0% when differentiating these activities at the first stage In addition, there is an obvious misclassification for Sitting and other activities attributed to the physical location of the device and our result of coarse classification is the essential steps to discrimination of these non-moving activities. At the second-stage CHMMs, we distinguished the special activities of walking and laying. This reduced the recognition burden for the third-stage. Based on the studies at the previous two stages, the accurate recognition is more simple which are only two activities. Through the seamless convergence of this three stages, the recognition rate for each activity has been significantly improved.

Although the use of accelerometer and gyroscope sensor could enhance the performance of the combination algorithm, it is unrealistic to believe that the general public will uninterruptedly perform the different kind of activities because of the the complex patternsof daily life, which is the daily behavior habits intergrating the moving activity and stationary activity. In our experiment, we only used Gyro-based data to recognize the activities attributed to the property of Sitting-Standing subclass. Thus, we repute that the method can reduce the complexity of the experiment and improve the accuracy of recognition. Since the smartphone is always sharing the information and services with other applications, this is advantageous so that we can perform the same study with the single specialized device in the future.

## Conclusion

In recent years, HAR of smartphone sensor data has received much attention. Generally, HAR of smartphone sensor data has its distinct advantages: firstly, it can continously record information of the subjects; secondly, it is cheap and convinent for ordinary people to use this type of HAR. We think that HAR of smartphone sensor data could be helpful for physical health management of elderly people living alone. Furthermore, we can imagine that it could also be helpful for psychological healthmanagement. However, these applications need to get high accuracy of HAR. If HAR of sensor data has low accuracy, these high level applicatons based on human activities would have higher error rate, such as psychological healthmanagement. The three-stage continuous hidden Markov model (TSCHMM) approach want to recognize the general activities on smartphone sensor data with high accuracy. As we know, CHMMs are professional to deal with the time-series data, such as accelerometer and gyroscope sensor data. On the other hand, human activities can usually be processed as structure objectives, which can help us design a multi-level classifier. As has been argued, the TSCHMM includes these ideas. In the expreiments mentioned above, the third-stage structure obviously reduced the number of features, thereby reduced the time complexity and saved the memory space. We also showed that the feature reduction technique of sparse local preserving projections combined with the feature selection method of random forest variable importance measures and other domain knowledge is effective in discovering the most available features.

Overall, our algorithms have achieved great performance. But for the future research, we still need to do more. There is much room to find a more effective method for feature selection and feature reduction. Analyzing the essential attribute of the obtained feature thoroughly can help to reduce the space and time complexity. What’s more, under the rapid development of cyber-physical sytems and the Internet of things, there are lots of new research topics with repect to body sensor data [26]. For instance, Lin *et al*. [27] applied the dynamic noise threshold technology to privacy protection, which may be more suitable for big data in body sensor network. Furthermore, it can also be a valuable research topic to develop the application which can online run on the smartphone platform with multimedia technology, such as touch-less interactive augmented reality game [28] and mobile health application based on virtual reality technology [29].
